# A Novel Multi-Dynamic Coupled Neural Mass Model of SSVEP

**DOI:** 10.3390/biomimetics10030171

**Published:** 2025-03-11

**Authors:** Hongqi Li, Yujuan Wang, Peirong Fu

**Affiliations:** 1 School of Software, Northwestern Polytechnical University, Xi’an 710072, China; wangyujuan12@mail.nwpu.edu.cn; 2 Yangtze River Delta Research Institute, Northwestern Polytechnical University, Taicang 215400, China; 3 Huawei Technologies Co., Ltd., Shenzhen 310051, China; 18406430685@163.com

**Keywords:** SSVEP modeling, BCI, neural mass model, PSO, dual-region coupling

## Abstract

Steady-state visual evoked potential (SSVEP)-based brain—computer interfaces (BCIs) leverage high-speed neural synchronization to visual flicker stimuli for efficient device control. While SSVEP-BCIs minimize user training requirements, their dependence on physical EEG recordings introduces challenges, such as inter-subject variability, signal instability, and experimental complexity. To overcome these limitations, this study proposes a novel neural mass model for SSVEP simulation by integrating frequency response characteristics with dual-region coupling mechanisms. Specific parallel linear transformation functions were designed based on SSVEP frequency responses, and weight coefficient matrices were determined according to the frequency band energy distribution under different visual stimulation frequencies in the pre-recorded SSVEP signals. A coupled neural mass model was constructed by establishing connections between occipital and parietal regions, with parameters optimized through particle swarm optimization to accommodate individual differences and neuronal density variations. Experimental results demonstrate that the model achieved a high-precision simulation of real SSVEP signals across multiple stimulation frequencies (10 Hz, 11 Hz, and 12 Hz), with maximum errors decreasing from 2.2861 to 0.8430 as frequency increased. The effectiveness of the model was further validated through the real-time control of an Arduino car, where simulated SSVEP signals were successfully classified by the advanced FPF-net model and mapped to control commands. This research not only advances our understanding of SSVEP neural mechanisms but also releases the user from the brain-controlled coupling system, thus providing a practical framework for developing more efficient and reliable BCI-based systems.

## 1. Introduction

Brain–Computer Interface (BCI) technology, as a cutting-edge research direction in neural engineering, provides direct real-time communication and control channels between users and external devices. By decoding brain electrical activities and converting them into device operation commands, BCI systems not only provide novel interaction methods for patients with severe motor disabilities but also pioneer new pathways in human–computer interaction. Among various BCI paradigms, non-invasive methods, particularly those based on electroencephalography (EEG), have garnered widespread attention due to their safety and portability [1].

EEG-based BCI systems utilize both spontaneous brain activity (endogenous signals) and stimulus-evoked responses (exogenous signals). While endogenous signals like Motor Imagery (MI) reflect more spontaneous internal cognitive processes [2], steady-state visual evoked potentials (SSVEPs) represent a powerful class of exogenous signals that offer distinct advantages [3]. SSVEPs are generated when users focus on visual stimuli flickering at specific frequencies, producing neural responses that precisely match these frequencies [4]. This frequency-specific characteristic enables reliable signal detection and efficient information encoding through multiple stimulation frequencies. The stability and robustness of SSVEP signals have led to their successful implementation in various applications, including brain-controlled robots [5,6], prosthetic systems [7], the five-digit robotic hand [4], and intelligent wheelchairs [8].

However, SSVEP-BCI development faces several challenges: (1) traditional SSVEP acquisition requires specialized EEG equipment, where the conductive gel is generally required in the wet device, increasing operational costs and limiting technological accessibility; (2) prolonged exposure to flickering stimuli induces visual fatigue, which may exhaust the engaged users; (3) signal quality and stability are affected by individual differences and environmental noise; and (4) the understanding of SSVEP signal physiological mechanisms through simulations remains incomplete due to the lack of relevant high-fidelity models. These issues impact system practicality, especially in the early prototype validation of certain SSVEP-based intelligent systems, and increase the complexity of signal processing and classification algorithm design.

Model simulations that produce virtual signals similar to actual EEG can be used to test and optimize SSVEP-BCI systems without the need for actual subject participation. Based on this, researchers can more rapidly evaluate the performance of the system, test different algorithms and parameter settings, and validate new SSVEP-BCI designs without the constraints of field experiments. This approach not only reduces research costs and reliance on experimental participants but also improves research reproducibility. Meanwhile, through simulation modelling, researchers are able to conduct various experiments and scenarios more flexibly, further promoting the development and application of SSVEP-BCI technology.

In fact, there are some studies that concentrate on SSVEP modeling. In the development of a neural mass model, Jansen’s pioneering work first demonstrated that mathematical models could successfully reproduce visual evoked potential (VEP) waveforms and revealed the crucial role of excitatory connections within the cortex [9]. Subsequently, the dual-channel coupled neural mass model developed by Jansen and Rit successfully simulated interactions both within and between cortical regions, generating EEG signals and visual evoked potentials highly consistent with actual observations [10]. Wendling et al. further extended this framework to a multi-channel coupled system, capable of simulating both low-frequency components (δ, θ, α) and high-frequency rhythms (β, γ) [11,12,13]. David and Friston’s research on dual-dynamic dual-channel coupled models elucidated the mechanisms of frequency modulation through excitatory/inhibitory parameters, inter-channel coupling strength, and time delays [14]. In conclusion, the traditional Jansen–Rit model employs a single set of excitatory and inhibitory linear transfer functions to describe neural population dynamics, and significant progress has been made in EEG signal analysis through both David and Friston’s dual-dynamic model and Cui’s multi-dynamic multi-channel coupled model [15]. Nevertheless, these models primarily focus on simulating general EEG activity, to our knowledge, no existing research has addressed the modeling of SSVEP based on the neural mass model (NMM) for practical applications.

To reduce dependence on physical EEG acquisition, enhance SSVEP understanding, and facilitate device control, this study proposes an innovative SSVEP neural mass modeling approach. The main contributions of our current study are depicted below.

1. Develop a bidirectionally coupled neural mass model of SSVEP, for the first time, to simulate cross-regional brain interactions. The developed multi-dynamic coupled NMM helps to elucidate the generation mechanisms of SSVEP signals.
2. Design parallel linear transfer functions and weight coefficient matrices for multiple stimulation frequencies. The entailed key parameters are further identified with the optimization using particle swarm algorithms.
3. Implement various data augmentation methods (signal translation, amplitude distortion, temporal masking, scale transformation, and noise addition). Utilize augmented real datasets to train high-performance SSVEP classifiers that can be used with advanced deep learning methods.
4. The application of advanced FPF-net classification algorithms for simulated signal recognition, with the further successful implementation on vehicle control.

This paper is organized as follows: Section 2 introduces the multi-dynamic coupled neural mass model for SSVEP-BCI, oscillator weight identification, and model parameter optimization. Section 3 presents the simulation analysis based on optimized parameters, classifier training for simulated SSVEP signals, and vehicle control implementation. Section 4 provides further discussion and conclusions.

## 2. Multi-Dynamic Coupled Neural Mass Model

Various human functions, such as thinking, perception, emotion, memory, and movement, are achieved by the interactions between neurons and the transmission of electrical signals. Neurons are the basic building blocks of the nervous system and usually include structures such as cell bodies, dendrites, and axons. A cluster of neurons is a functional grouping of multiple neurons in the nervous system formed by synaptic connections. These groups of neurons usually work together in performing specific neural activities or processing specific information.

The EEG, recorded via head epidermal electrodes, reflects the macroscopic activity of neuronal populations, which is a composite of the postsynaptic currents of a large number of neuronal populations in the brain tissue, either in the cerebral cortex or on the surface of the scalp. Thus, it is possible to model EEG signals from a computational macroscopic model of neuron populations, which is termed as the neural mass model.

### 2.1. Traditional Neural Mass Model

The neural mass model represents the average behavior of large neuronal populations rather than individual neurons, which is particularly suitable for studying large-scale brain dynamics, as observed in EEG recordings. The traditional neural mass model incorporates three distinct neuronal populations: pyramidal neurons, excitatory interneurons, and inhibitory interneurons.

More specifically, pyramidal neurons are the principal cells responsible for transmitting the main output from the cortex. In addition to these, excitatory interneurons play a crucial role in enhancing neural activity within localized regions, while inhibitory interneurons function to suppress neural activity, maintaining a balance within the neural circuits. These different types of neurons work together to regulate the overall activity of the cortical network.

Signal processing within each population involves two primary transformations. The first is somatic transformation, where the average membrane potential is converted to the mean firing rate through the sigmoid function *s*(*v*) at the neuron soma [16], which can be described as

(1) $$s\left(v\right)=\frac{2e_{0}}{1+e^{\left(v_{0}-v\right)}}$$

where 2*e*_0_ determines the maximum firing rate of the neuronal population, *r* represents the steepness of the sigmoid function reflecting the sensitivity of the population response, *v*_0_ means the postsynaptic membrane potential at the firing rate of *e*_0_, and *v* is the inputted average presynaptic membrane potential.

The second is synaptic transformation, occurring at neuronal synapses, where presynaptic firing rates are converted into postsynaptic potentials [17]. This process generates either excitatory or inhibitory responses, mathematically expressed as:

*h_e_*(*t*) = *u*(*t*)*G_e_ω_e_te^−tωe^* (2)

*h_i_*(*t*) = *u*(*t*)*G_i_ω_i_te^−tωi^* (3)

**Figure 1:**
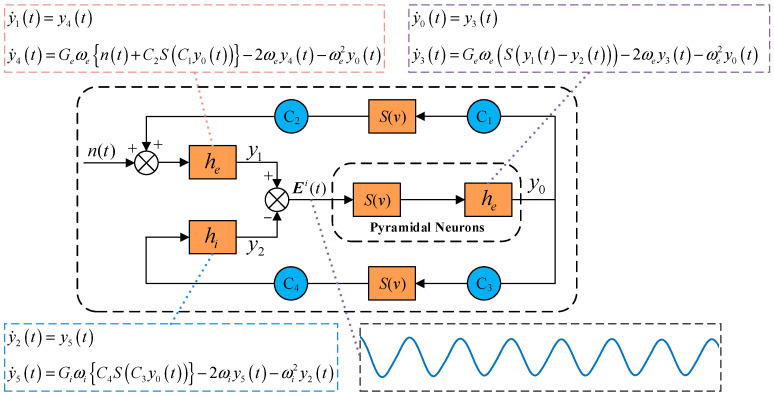
The traditional neural mass model, which contains excitatory interneurons, inhibitory interneurons, and pyramidal neurons. A sigmoid function *S*(*v*) and differential equations for excitatory (*h_e_*) and inhibitory (*h_i_*) responses are included to describe the dynamic behavior of the interested subpopulation. The external input *n(t)* is modeled as Gaussian white noise, which introduces variability to the signal, and the coupling coefficients *C*_1_, *C*_2_, *C*_3_, and *C*_4_ define the interaction strengths between different neural subpopulations. The output signal *E^i^(t)*, being the difference between the excitatory and inhibitory responses, represents the EEG-like signal produced by the model.

Through the Laplace transformation of *h*(*t*) and subsequent inverse transformation, we derive two ordinary differential equations for each subpopulation (see in Figure 1). In this framework, *y*_0_(*t*), *y*_1_(*t*), and *y*_2_(*t*) denote the postsynaptic membrane potential outputs from the pyramidal, excitatory interneuron, and inhibitory interneuron subpopulations, respectively. Their corresponding first derivatives are represented by *y*_3_(*t*), *y*_4_(*t*), and *y*_5_(*t*). The model parameters *G_e_* and *ω_e_* represent the excitatory average synaptic gain and inverse time constant, while *G_i_* and *ω_i_* denote their inhibitory counterparts. External inputs and undefined regional signals are modeled as Gaussian white noise, represented by *n*(*t*), which can also be expressed as *N(μ, σ^2^)*, where *μ* represents the mean and *σ*^2^ represents the variance. The connectivity strengths between pyramidal neurons and interneuron subpopulations are quantified by the coefficients *C*_1_, *C*_2_, *C*_3_, and *C*_4_. The model generates an EEG-like output signal computed as the difference between excitatory and inhibitory interneuron subpopulation responses *E(t)* = *y_1_*(*t*) − *y_2_*(*t*).

As shown in Figure 1, the traditional NMM can only represent single-cell population dynamics, generating narrowband signals at specific frequencies. However, actual EEG signals exhibit broad spectral characteristics, with different rhythms reflecting various brain behaviors. In order to simulate real SSVEP signals with a wider spectrum, SSVEP-BCI multi-dynamic neuron mass models need to be built so that the models can exhibit multiple sets of excitatory and inhibitory dynamics to represent multiple types of cellular subpopulations in the neuronal network. David established a dual-dynamic NMM [14], which Cui later extended into a multi-dynamic NMM [15]. For these models, each neuronal population comprises multiple neuronal subgroups with distinct dynamic characteristics, represented through parallel linear transfer functions. Weight parameters *w_i_ (*0 *≤ w_i_ ≤* 1, Σ*w_i_ =* 1*)* were introduced to modulate the relative contributions of neuronal subgroups with different dynamic characteristics. In our current work, the actual SSVEP signals are divided into several sub-bands and the power spectral density of the three neuronal subpopulations of *δ*, *α*, and *γ* are calculated to determine the corresponding weight coefficients. The detailed design of our model is as follows.

### 2.2. Multi-Dynamic Coupled NMM for SSVEP-BCI

Experimental studies on SSVEP response characteristics that are widely used in the SSVEP-BCI based intelligent systems indicate that stimulation frequencies typically range from 4 to 50 Hz. Therefore, we divided the aimed EEG power spectral density into five sub-bands: 0–4 Hz, 4–8 Hz, 8–16 Hz, 16–32 Hz, and 32–64 Hz. Furthermore, considering both the SSVEP response intensity and spectral coverage requirements, three key primary sub-bands that can be generated by the above traditional NMM were selected:

- *δ* wave (0–4 Hz): characterizing low-frequency response properties;
- *α* wave (8–16 Hz): corresponding to the peak SSVEP response interval;
- *γ* wave (32–64 Hz): capturing high-frequency dynamics.

Therefore, a hypothesis was defined in our current developed model; that is, the SSVEP EEG signal for any desired rhythm can be derived from the simulation of a dynamic combination of the selected three primary sub-band signals described above. In the proposed multi-dynamic neural mass model for SSVEPs, each neuronal subpopulation incorporates three parallel linear transfer functions: *h_e_*^δ^, *h_e_*^α^, and *h_e_*^γ^ for excitatory responses and *h_i_*^δ^, *h_i_*^α^, and *h_i_*^γ^ for inhibitory responses. These characteristics are modulated through weights *ω*^δ^, *ω*^α^, and *ω*^γ^, enabling the precise modeling of characteristic frequency bands through optimized weighted combinations, while maintaining constant synaptic connectivity coefficients (*C*_1_*–C*_4_) to ensure biological plausibility. More specifically, compared with the model shown in Figure 1, the proposed SSVEP-BCI multi-dynamic neural mass model extends the dynamic linear transformation process from a single linear transformation function to a parallel weight adjustment by three types of linear transformation functions, which directly affects the inputs and outputs of different neuron populations in the model, and thus changes the dynamic linear transformation process.

**Figure 2:**
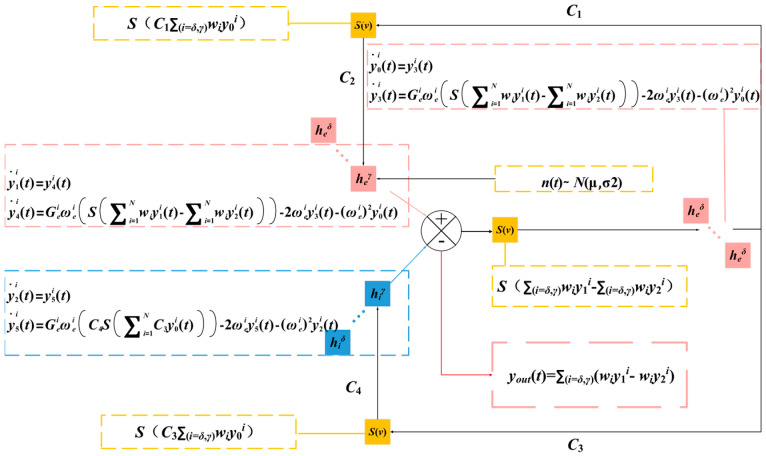
The multi-dynamic neural mass model for SSVEPs.

Figure 2 illustrates the information transformation process of the SSVEP-BCI multi-dynamic neural mass model, where *y_out_* represents the regional output.

The brain is a highly differentiated and complex organ, consisting of many different brain regions, each with a specific function, and the model in Figure 2 is able to yield output from certain regions. However, since SSVEP-based studies encompass complex cognitive processes, such as attention, memory, and decision-making, these processes involve the visual cortex of the brain. Included in the visual cortex are areas partially located in the occipital and parietal regions, with occipital regions being the main visual processing areas and parietal regions being related to some extent to the processing and integration of visual information. As a result, signal generation in SSVEP involves interactions between the occipital and parietal regions, so it is more important to take into account the coupling between the brain areas in this case [18].

**Figure 3:**
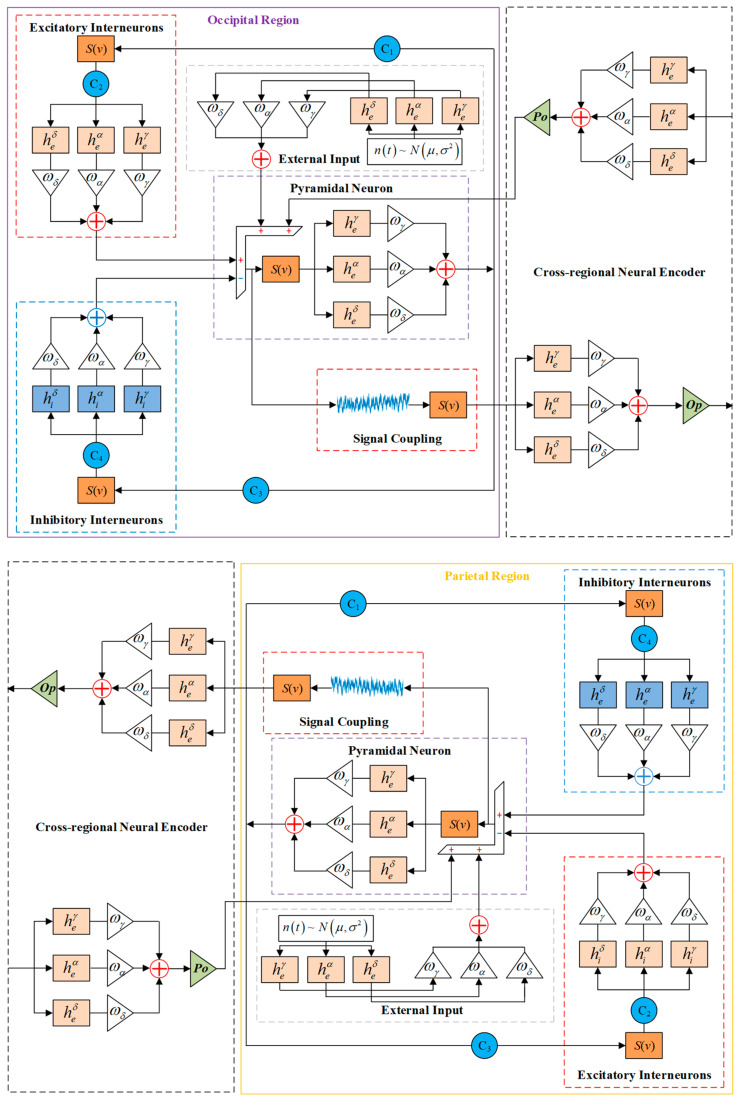
The SSVEP-BCI multi-dynamic coupled neural mass model. The occipital and parietal regions are represented by a multi-dynamic NMM of Figure 2, where three parallel linear transfer functions are involved in the excitatory and inhibitory interneurons. The membrane potential of each intra-regional pyramidal cell (i.e., *y_out_*) is first transformed into mean spike density through the static nonlinear function *s*(*v*) and then processed by the cross-regional neural encoder.

Cross-regional information transfer is primarily achieved through the pyramidal cells’ long-range axonal projections. During information transfer, the membrane potential of intra-regional pyramidal cells (*y_out_*) is first transformed into mean spike density through the static nonlinear function, *s*(*v*), then processed by the cross-regional neural encoder. The cross-regional neural encoder is also represented by three weighted parallel transfer functions, ultimately transmitting the processed signal as coupling signals to other regions. The model introduces coupling coefficients *o_p_* (from occipital to parietal) and *p_o_* (from parietal to occipital) to characterize bidirectional interactions. To construct the multi-dynamic coupling model, we coupled two SSVEP-BCI multi-dynamic neural mass models representing the parietal and occipital regions, as illustrated in Figure 3.

Moreover, the *So_p_* denotes the coupling signal transmitted from the occipital to parietal region, then we modelled the *So_p_* as:

(4) $$S_{O_{P}}=O_{P}\cdot \mathrm{RM}\left(S\left(\sum_{i=\delta }^{\gamma }\omega_{oi}y_{1\omega_{\tau }^{-1}}^{oi}-\sum_{i=\delta }^{\gamma }\omega_{oi}y_{2\omega_{\tau }^{-1}}^{oi}\right)\right)$$

where we used the de-mean function *RM(x) = x-mean(x)* to perform the de-mean operation, *S*(·) is the static nonlinear function of Equation (1), *ω_oi_* represents the weights of oscillatory modulators with different frequency components (e.g., δ (0–4 Hz), α (8–16 Hz), and γ (32–64 Hz)) in the occipital region, *y^oi^* means the post-synaptic membrane potential in the occipital area after each of the linear transformation functions with different dynamic behaviors, that is, *y*_1_ and *y*_2_ denote the postsynaptic membrane potential of the excitatory and inhibitory interneuron populations, respectively. *i* = *δ*, *α*, *γ* denotes the three subpopulations of neurons placed side-by-side with different dynamic behaviors.

The coupling signal *So_p_* reflects the net excitatory–inhibitory balance in the occipital region, weighted by frequency-specific contributions (δ, α, γ). This balance modulates parietal region activity, simulating how visual processing areas influence higher-order cognitive regions during SSVEP generation.

So far, we have completed the construction of a dynamic model of the NMM for the relevant SSVEP, from which it is expected that all desired signals can be simulated and output.

### 2.3. Weight Parameter Identification for Oscillators

Due to the frequency characteristics of SSVEP signals changing with visual stimuli, using anatomically based empirical parameters that lack dynamic characteristics fails to reflect the specific behavior of SSVEP signals at different frequencies. Furthermore, in real SSVEP-BCI experiments, individual neural structural differences among subjects can result in significant variations in the dynamic properties of the occipital and parietal regions. These regions may exhibit different sensitivities to the visual stimuli of various frequencies, leading to substantial differences in the evoked SSVEP signals. Therefore, for each subject and for signals at different frequencies, it is necessary to identify the multi-dynamic behavior of these two brain regions.

Considering the visual stimulation characteristics of SSVEP, different visual stimulus frequencies generate SSVEP signals of varying frequencies. Specifically, under different visual stimulation frequencies, the EEG sub-bands should exhibit distinct energy distribution characteristics. These energy ratios can then be used to reverse engineer the weight coefficients of the linear transformation functions.

The real EEG signals are transformed into spectral information using the Fast Fourier Transform (FFT) to obtain their power spectral density (PSD). To further explore brain activity characteristics in different frequency ranges, the PSD is divided into five critical sub-bands: δ band (0–4 Hz), θ band (4–8 Hz), α band (8–16 Hz), β band (16–32 Hz), and γ band (32–64 Hz). Based on the significant physiological relevance of SSVEP neurodynamic modeling, here the emphasis is placed on the δ, α, and γ bands. The energy ratios of the δ, α, and γ rhythmic waves within their respective sub-bands to the total energy of the three sub-bands are then calculated as the weight coefficients ω. On the basis of the above model assumptions, the weight coefficients must satisfy two fundamental constraints: the value of each component must range between 0 and 1, and their total sum must be equal to 1.

Given the significant dynamic differences between the cortical regions and the lack of reliable empirical values for key model parameters (such as cross-region coupling coefficients and the mean and variance of external Gaussian white noise inputs) in current anatomical studies, one effective optimization algorithm is applied to systematically optimize the model parameters. According to the parameter analysis of the basic neuron population model, the excitatory mean synaptic connectivity *C*_1_ and *C*_2_, the inhibitory mean synaptic connectivity *C*_3_ and *C*_4_, and the parameters *v*_0_, *e*_0_, and *r* in the static nonlinear function have a clear physiological significance and do not change according to the generation of the underlying waveforms, such as δ, α, and γ, so that the empirical values of the parameters are maintained unchanged.

The parameters to be optimized include: the coupling coefficient from the occipital to the parietal region (*o_p_*), the coupling coefficient from the parietal to the occipital region (*p_o_*), and the mean (*μ*) and variance (*σ^2^*) of Gaussian white noise for each region.

### 2.4. Particle Swarm Optimization-Based Optimization for Identified Parameters

Particle Swarm Optimization (PSO) is a population-based optimization algorithm inspired by the collective behavior of natural organisms, such as bird flocks and fish schools [19]. The algorithm seeks optimal solutions by simulating the search process of individuals (particles) in the solution space. The steps are as follows:

1. Particle Representation and Initialization: Each particle is represented by a position vector in the solution space, and its velocity vector defines the direction and rate of search. For the i-th particle, its position is represented as *X_i_ = (x_i1_, x_i2_, …, x_in_)*, and its velocity as *V_i_ = (v_i1_*, *v_i2_*, *…*, *v_in_)*.
2. Objective Function Definition: The Mean Squared Error (MSE) is used as the objective function to measure the difference between the model’s predicted frequency spectrum and the real collected frequency spectrum:

(5) $$MSE=\frac{1}{n}\sum_{i=1}^{n}{\left[Y\left(f_{i}\right)-S\left(f_{i};\theta \right)\right]}^{2}$$

where *i* ranges from 1 to *n*; *n* represents the total number of sampling points; *Y*(*f_i_*) represents the spectrum of the real SSVEP signal; *S(f_i_;θ)* represents the spectrum of the simulated SSVEP signal by the multi-dynamic neural mass model; *f* represents frequency; and *θ* represents the parameters to be optimized in the model. A smaller MSE indicates better model fitting.

3. Particle Velocity and Position Update: Each particle updates its velocity and position according to the following formulas:

(6) $$V_{ij}^{t+1}=kV_{ij}^{t}+c_{1}\times r_{1}\times \left(pbest_{ij}-X_{ij}^{t}\right)+c_{2}\times r_{2}\times \left(gbest-X_{ij}^{t}\right),$$

(7) $$X_{ij}^{t+1}=X_{ij}^{t}+V_{ij}^{t}$$

where *i* ∈ [1, 50] represents particles; *t* means the iteration number, with a total of 70 iterations; *j* is the dimension of each particle, where the number of parameters to be optimized in the model is 18; *V_ij_^t^*^+1^ represents the velocity update for particle *i* in dimension *j*; *X_ij_^t+^*^1^ is the position update for particle *i* in dimension *j*. *k* represents the inertia weight, set to 0.5, used to retain a portion of the previous velocity; *c*_1_ is the individual learning factor, meaning the extent to which each particle adjusts its velocity and position based on its own historical experience, set to 1.5; *c*_2_ is the social learning factor, indicating the extent to which each particle adjusts its velocity and position based on the entire swarm’s historical experience, set to 1.5, ensuring that *c*_1_ *+ c*_2_ falls between 0 and 4; *r*_1_ and *r*_2_ are random numbers between 0 and 1; *pbest_ij_* represents the historical best position of particle *i* in dimension *j*; and *gbest_j_* represents the global best position across the entire swarm in dimension *j*.

4. Update of Individual and Global Best Positions: Each particle dynamically updates its personal best (*pbest*) by retaining the parameter combination (position) that yields the lowest MSE value encountered during its search history. The swarm collaboratively identifies the global best (*gbest*)—i.e., the single position across all 50 particles that achieves the minimum MSE throughout the optimization process.
5. Termination Condition: The PSO terminates when the preset number of iterations (t = 70) is reached.

To ensure the reasonable movement of particles within the search space, the upper and lower bounds for each parameter (dimension) must be defined. The bounds for the mean (*μ*) of the external Gaussian white noise are set as [22, 2200], and the bounds for the variance (*σ^2^*) are set as [2, 20,000].

## 3. Results

### 3.1. Simulation of SSVEP-BCI Multi-Dynamic Coupled Neural Mass Model

**Figure 4:**
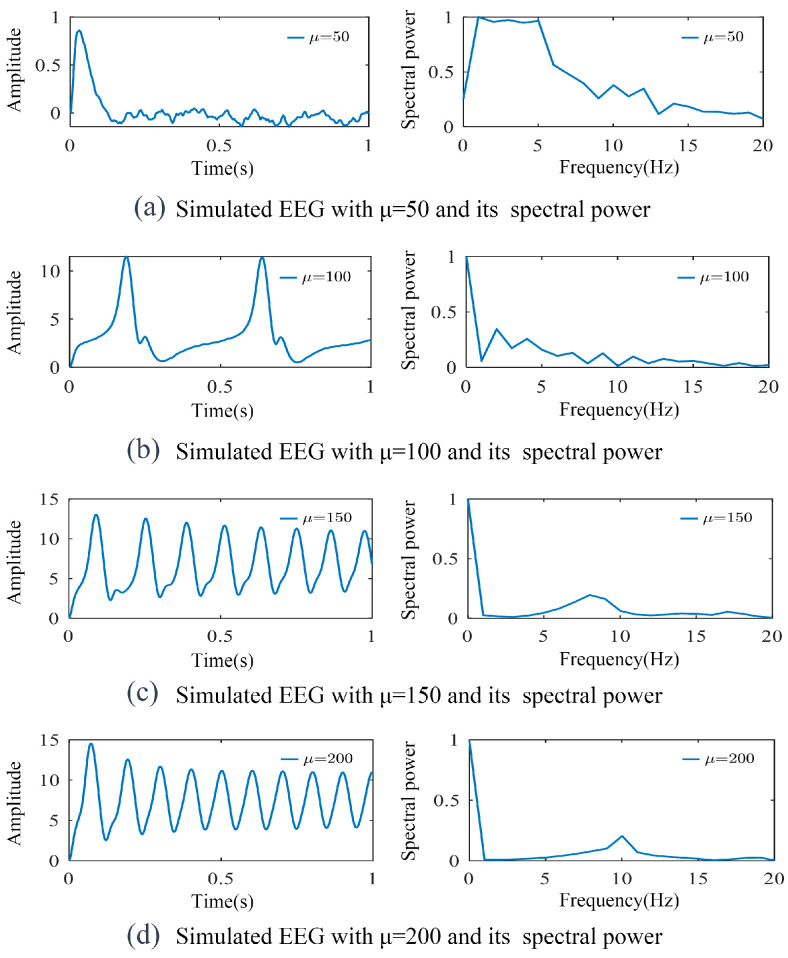
Simulated signal curves varying with *μ* and their spectral power. As *μ* increased from 50 to 200, the rhythmic characteristics gradually intensified, with a final pronounced spectral peak at 10 Hz.

In investigating the effects of the model parameters on output characteristics, we first studied the influence of the external input parameters using the traditional NMM, with a baseline sampling time of 4/4096 s and fixed parameters: excitatory synaptic gain *G_e_* = 3.25 mV, inverse of excitatory time constant *ω_e_* = 100 s^−1^, inhibitory synaptic gain *G_i_* = 22 mV, and inverse of inhibitory time constant *ω_i_* = 50 s^−1^. As shown in Figure 4, varying the mean external input *μ* from 50 to 200 revealed distinct signal characteristics. The variation of *μ* reflects the adjustment of the external stimulus intensity. At *μ* = 50, sporadic spikes with predominantly low-frequency components emerged. When *μ* is low, sparse spikes dominated by low-frequency components emerge, which may correspond to the brain’s activity pattern during rest or low activation states. As *μ* increases (to 100 and 150), regular spikes with frequencies concentrated between 1 and 11 Hz appear, which may align with the brain entering a higher activation state, preparing to respond to external stimuli. At *μ* = 200, the rhythmic features strengthened with a pronounced spectral peak at 10 Hz.

**Figure 5:**
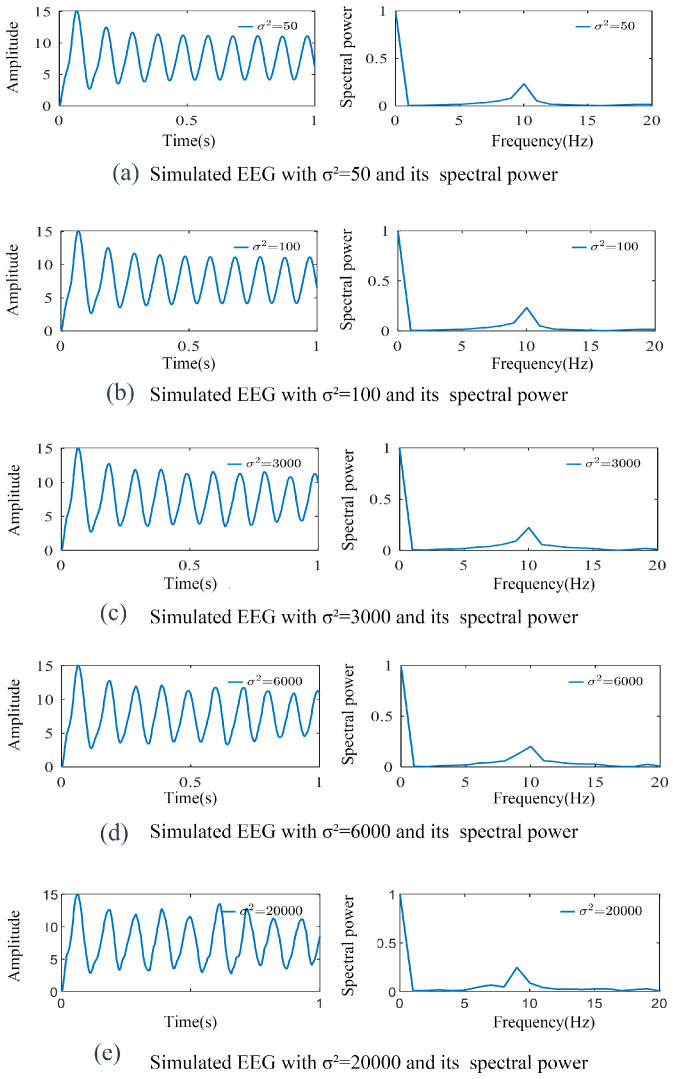
Simulated signal curves varying with *σ*^2^ when *μ* = 220 and their spectral power. As *σ^2^* increased from 50 to 20,000, slight to progressive changes of amplitude and spectral peaks variations were observed.

The variance *σ^2^* represents the level of noise or randomness in external inputs, affecting neural network responses and synchronization. The effects of varying *σ*^2^ (with *μ* = 220 and other parameters held constant) are illustrated in Figure 5. As we can see, at lower σ^2^ values of 50–100 (indicating the relatively stable external input), a strong oscillatory pattern in neural activity, characterized by stable 10 Hz alpha wave features, is observed. This may correspond to brain behavior during relaxed states, similar to how SSVEP signals exhibit stable oscillations during resting conditions, which enhances sensitivity to stimulus frequencies. As *σ*^2^ increases to 3000, although oscillation amplitudes show slight variations, the spectral profile still maintains its stability, demonstrating the system’s adaptability to moderate noise levels. As such, the biomimetic behavior mirrors the brain’s ability to maintain SSVEP signal stability under moderate stimulation intensities, reflecting the neural mechanisms for maintaining synchronized oscillations despite mild environmental perturbations. Further increases in *σ*^2^ (i.e., to 6000 and even 20,000) lead to progressive changes in the spectral peaks and rhythmic patterns. This phenomenon parallels brain activity under high cognitive load or stress conditions, where neural activity becomes more irregular and susceptible to perturbations. Under these conditions, SSVEP signal stability may be compromised, manifesting as more dispersed frequency distributions and reduced synchronicity, similar to the brain’s stress response to complex or unpredictable stimuli.

**Figure 6:**
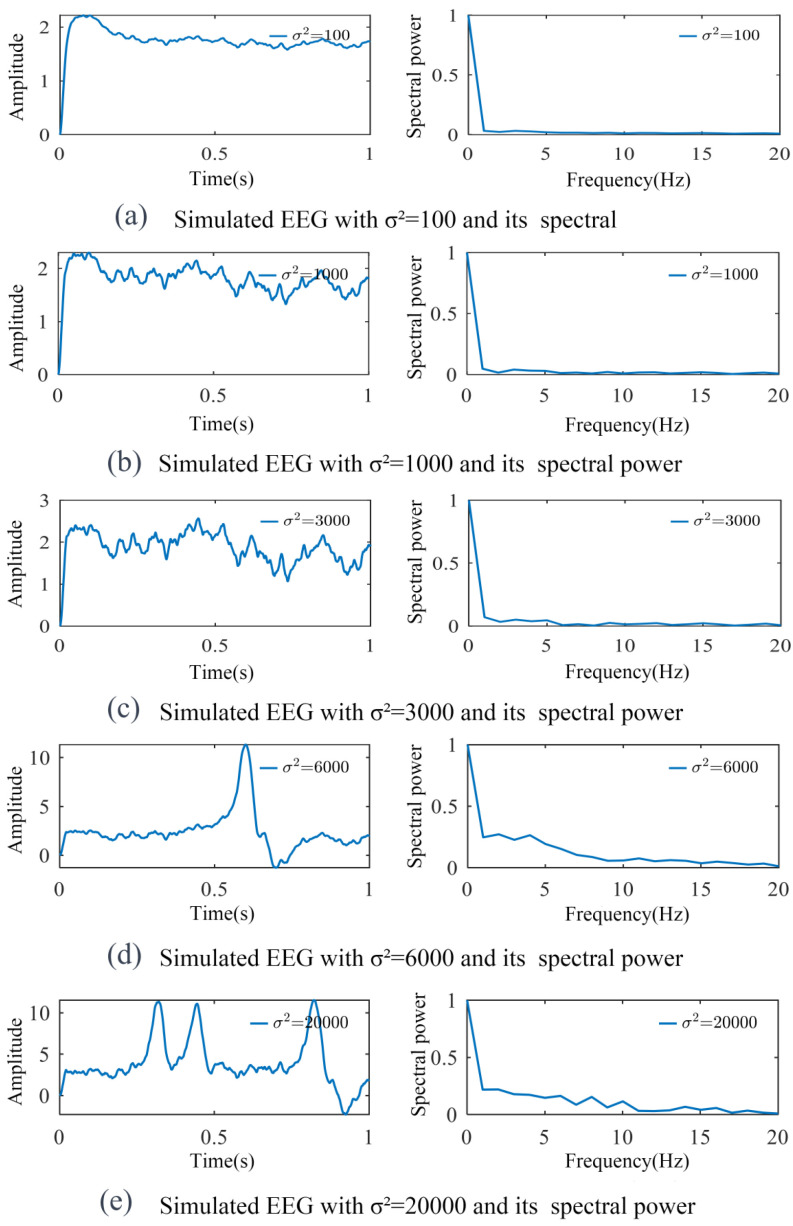
Simulated signal curves varying with *σ*^2^ when *μ* = 90 and their normalized spectral power. As *σ*^2^ increased, signal amplitudes gradually increased (e.g., from 100 to 3000), and even led to irregular spike activity (from 6000 or 20,000).

Under low *μ* conditions, representing weaker external stimulation and potentially lower brain activation states, noise effects become more pronounced. In this regard, when *μ* is reduced to 90 while keeping other parameters constant, the model exhibits enhanced sensitivity to *σ*^2^ variations, as shown in Figure 6. Specifically, as *σ^2^* increases from 100 to 3000, signal amplitudes gradually increase, manifesting as stronger oscillations. Further increases in *σ*^2^ to 6000 or 20,000 leads to the transformation of regular oscillations into irregular spike activity. This biomimetic response pattern may indicate neural dynamics during weak or unstable stimulation conditions, where neural activity becomes more chaotic and less predictable.

**Figure 7:**
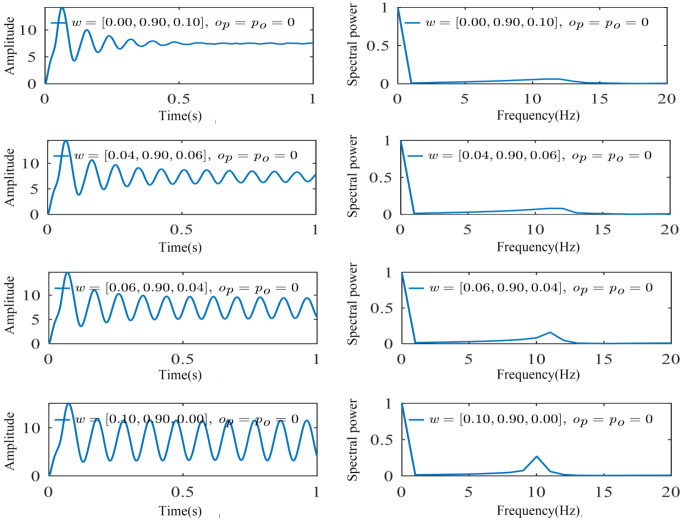
The simulated signals and spectral power of the occipital region without coupling. Due to the high weight assigned to α, the waveform fluctuated around the alpha wave, and as the delta wave component increased, spike activity decreased with a gradual left-ward shift in frequency peaks.

When studying the influence of ω on the multi-dynamic behavior of neural systems, adjustments to *ω* represent the relative contributions of different frequency bands (such as *α*, *δ*, and *γ* waves) between brain regions, thereby affecting the overall pattern of neural oscillations. In this study, we employed the decoupling model shown in Figure 2 and fixed the weight coefficient *ω^α^* of the alpha oscillator at 0.90 in both occipital and parietal neuronal populations while successively increasing the weight *ω^δ^* of the delta oscillator and decreasing the weight ω^γ^ of the gamma oscillator. As shown in Figure 7, due to the high weight assigned to α, the waveform fluctuated around the alpha wave, indicating that the brain was in a relaxed, resting state. Under this state, the neural activity is highly synchronized, exhibiting typical low-frequency activity patterns. As the weight of the *δ* wave increased and the weight of the *γ* wave decreased, sharp peak activities within the system decreased, and the frequency peaks shifted towards lower frequencies.

**Figure 8:**
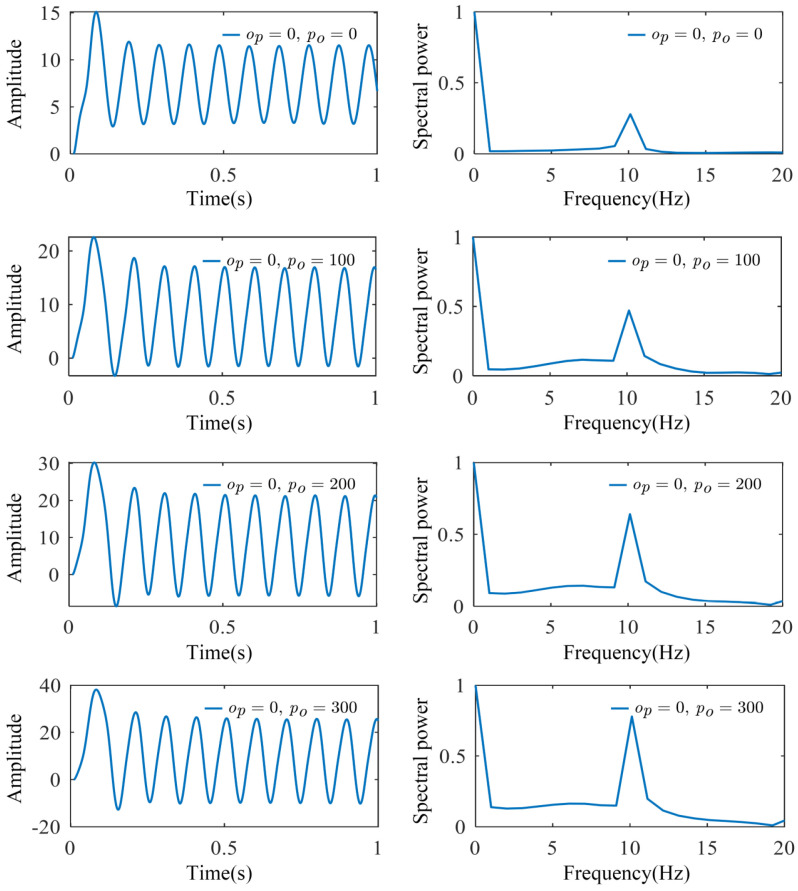
The simulated signals and spectra of the occipital region under unidirectional coupling. As the parietal-to-occipital coupling strength (*p_o_*) increased, while maintaining zero occipital-to-parietal coupling (*o_p_* = 0), the occipital region showed an increased signal amplitude while maintaining stable frequency characteristics, accompanied by enhanced spectral peak values.

When investigating the effects of regional coupling we used the model shown in Figure 3, where two regions exhibit the same dynamic characteristics (weight coefficient matrix *ω* = [0.10, 0.90, 0.00]) and there exists a unidirectional coupling from the parietal lobe to the occipital lobe. This model reflects the functional connectivity between different brain regions, particularly the regional interactions involved in processing visual information. By simulating the interactions between brain regions, we are able to uncover the neural network modulation mechanisms during specific cognitive tasks. As shown in Figure 8, increasing the coupling strength (*p_o_*) from the parietal lobe to the occipital lobe simulated an enhanced neural network when the brain perceives and processes visual stimuli. This led to a significant increase in the signal amplitude in the occipital lobe, indicating stronger visual processing activity. This suggests that the occipital region becomes more active during the processing of visual information, making the brain’s response to visual stimuli more sensitive. At the same time, the frequency characteristics remain stable, indicating that despite the increase in neural activity amplitude, the brain maintains efficient frequency regulation, ensuring the stability of cognitive functions. The enhanced spectral peak could be related to the increased involvement of the occipital region in visual processing, signaling greater focus and activity in this region during visual tasks. Moreover, by adjusting the coupling strength, we can simulate and analyze the coordination between different regions of the brain, providing deeper insights into the functional distribution and collaborative mechanisms across the brain areas.

**Figure 9:**
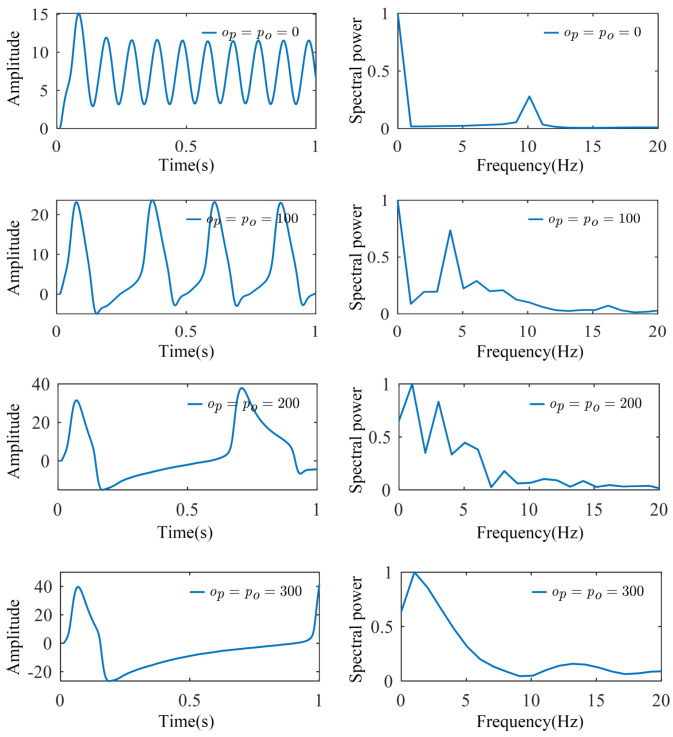
The simulated signals and spectra of the occipital region under bidirectional coupling with different dynamic characteristics. When the coupling strength between the regions increases, the occipital region model simulation signal spikes are reduced, and the spectral peaks are gradually shifted to the left.

Finally, we tested the case where the regions have different dynamic properties and are coupled in bidirections. The dynamic properties of the occipital and parietal regions were made different, i.e., the weight coefficients of the oscillatory modulators for each neuron population in the two regions were different, with the weight coefficient matrix of the occipital region fixed at *ω*_1_ = [0.10, 0.90, 0.00] and that of the parietal region fixed at *ω*_2_ = [0.40, 0.30, 0.30]. The occipital region and parietal region are coupled in both directions, and the coupling strength is the same, *o_p_* = *p_o_*. The EEG signals and normalized spectra of the occipital region under different coupling strengths are shown in Figure 9.

As can be seen in Figure 9, when the occipital region is coupled with the parietal region, which has a larger *ω^δ^* weighting coefficient, the occipital region model simulation signal spikes are reduced, and the spectral peaks are gradually shifted to the left as the coupling strength between the regions increases. Like the bidirectional coupling of regions with the same dynamic characteristics, the spectrum shows a bispectral peak at *o_p_* = *p_o_* = 200, which is then mainly concentrated in the low-frequency frequency domain.

Indeed, when the occipital and parietal regions were bidirectionally coupled, the greater *ω^δ^* weighting in the parietal region resulted in an amplified influence of the low-frequency delta waves on the occipital region. This, in turn, suppressed high-frequency activity in the occipital region, leading to a reduction in the signal peaks. As the coupling strength between the regions increased, this inhibitory effect became more pronounced, and the spectral peaks gradually shifted towards the lower frequency range, indicating a concentration of brain oscillatory activity in the low-frequency domain. In a scenario where both regions exhibited identical dynamic properties and were bidirectionally coupled, similar results were observed. When *o_p_* = *p_o_* = 200, the spectrum displayed a double peak, which then predominantly concentrated in the low-frequency range. The increase in coupling strength corresponds to an enhancement of the functional interaction between regions, thereby providing valuable insights into how the brain adjusts the functional allocation and information transfer between regions under different cognitive states.

These comprehensive simulations demonstrate the complex interplay between external input parameters (*μ* and *σ^2^*) and their significant influence on model output, with *σ^2^* requiring substantial changes to impact results. Furthermore, the weight coefficients and coupling strengths significantly affect both signal output and spectral characteristics. These findings emphasize the crucial importance of precise parameter identification for generating realistic SSVEP signals in the SSVEP-BCI multi-dynamic coupling model.

### 3.2. Parameter Identification and Model Validation

To validate the effectiveness of the SSVEP-BCI multi-dynamic coupled neural mass model, we analyzed SSVEP signals under 10 Hz, 11 Hz, and 12 Hz stimulation frequencies. The analysis of real SSVEP signals (Table 1) revealed consistent energy distribution patterns across frequency bands, with the γ band (32–64 Hz) showing dominant energy ratios (82.69–83.36%), followed by the α band (8–16 Hz) at 12.39–13.31%, and the δ band (0–4 Hz) contributing 3.84–4.59% across all stimulation frequencies. This distribution informed the weight coefficient matrix for the neural oscillators.

The PSO algorithm optimized six key model parameters, with Table 2 showing their optimized values across stimulation frequencies. The bidirectional coupling coefficients varied notably: *o_p_* ranged from 359.44 to 1566.06, while *p_o_* varied from 17.78 to 370.92. The Gaussian white noise parameters also showed distinct patterns across regions, with mean values (*μ*) spanning from 899.50 to 1741.10 and variances (*σ^2^*) ranging from 1709.47 to 17978.49.

**Figure 10:**
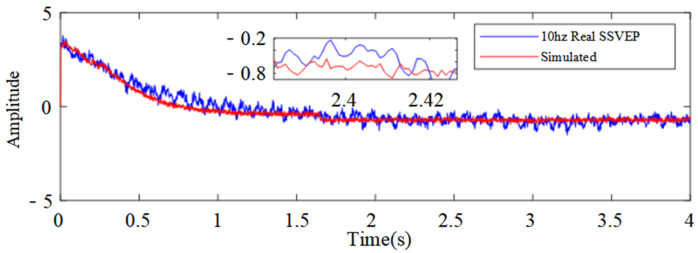

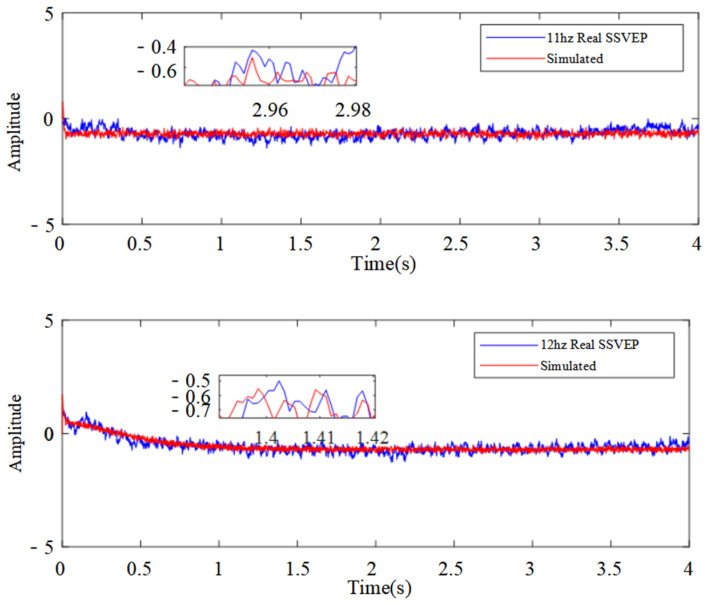
Comparison of real and simulated SSVEP under three types of visual stimuli, where overall waveform pattern of simulated signals remains consistent with real signals.

Model simulations using the optimized parameters demonstrated a strong alignment with real SSVEP signals (Figure 10). In a 10 Hz stimulation, while some amplitude variations occurred during the 0.5–1 s interval, the overall waveform pattern remained consistent with real signals. The 11 Hz simulations showed high accuracy throughout, with only slight deviations near 0.2 s. The 12 Hz simulations achieved the best performance, exhibiting optimal signal matching across the entire recording period in both waveform characteristics and phase information.

The quantitative performance evaluation using three error metrics (Table 3) showed a systematic improvement with increasing stimulation frequency. As frequency increased from 10 Hz to 12 Hz, MAE decreased from 0.3226 to 0.1520, RMSE from 0.4182 to 0.1906, and ME from 2.2861 to 0.8430. This pattern of improved accuracy at higher frequencies suggests an enhanced SSVEP response stability under high-frequency stimulation, demonstrating satisfactory model performance across all tested frequencies.

In the absence of an actual magnitude difference criterion, it is difficult to make a statement on the absolute superiority of the current model. Moreover, real SSVEP experiments are influenced by multiple complexities such as subject-related factors (eye movements, blinks), visual fatigue effects, and environmental variables (lighting, ambient noise, electromagnetic interference). Our model, using Gaussian white noise with fixed parameters and simplified physiological characteristics, cannot fully replicate these experimental complexities. Nevertheless, the same time-domain signal processing, including filtering, feature extraction, classification, and finally control on the external device, can be conducted to illustrate the usability of the model from a macroscopic point of view.

### 3.3. Simulated SSVEP Signal Processing and Classification

**Figure 11:**
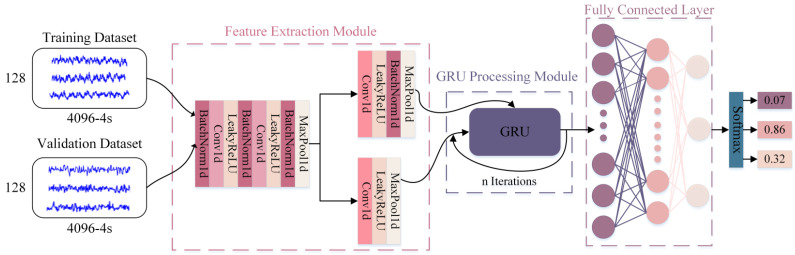
FPF-net structure.

To address the common issue of insufficient samples in SSVEP experimental data collection (e.g., only six recordings per target in the Benchmark dataset [20] and four in the Beta dataset [21]), we designed a systematic data augmentation strategy. Specifically, five augmentation methods were applied to each original signal: signal shifting, amplitude distortion, temporal masking, scale transformation, and noise addition. Each sample underwent 100 random augmentation operations, expanding the dataset to 27,000 samples. For the effective classification of simulated SSVEP signals, we proposed the FPF-net model, which integrates the Gated Recurrent Unit (GRU) and Convolutional Neural Network (CNN) [22]. The model processes 4096 sampling points through multi-level feature extraction modules for dimensionality reduction and feature extraction, utilizing GRU’s gating mechanism for selective information flow, updating and capturing long-term dependencies in sequential data through multiple iterations, as shown in Figure 11.

The model was implemented using the PyTorch 2.0 framework and trained on an NVIDIA GeForce RTX 3060 Laptop GPU. Training parameters were set as follows: batch size 128, initial learning rate 0.0001, Adam optimizer with weight decay 0.5×10^−4^, and cross-entropy loss function. To demonstrate the superiority of the proposed architecture, we compared FPF-net with various classical methods including Support Vector Machine (SVM) [23], Long Short-Term Memory (LSTM) [24], AttnSleep [25], and CMFnet [26]. For AttnSleep and CMFnet, we adjusted their fully connected layer input dimensions and modified their output to three classes. The experimental results showed that FPF-net achieved accuracy rates of 0.821 and 0.813 on training and validation sets, respectively, as shown in Table 4, which satisfies the accuracy requirement of online SSVEP-based device control [8].

To validate the model’s practical applicability, we designed a real-time control experiment using an Arduino car. A “7”-shaped track, including straight, left turn, and right turn paths, was set up in a real environment. We first obtained the relevant command sequences based on the predefined trajectories. Then, based on the command settings (10 Hz for left turn, 11 Hz for straight movement, and 12 Hz for right turn commands), the corresponding stimulus frequencies were obtained. The proposed model was used to obtain the corresponding EEG data by inputting different optimized physiological parameters and to be processed and classified. Finally, the practical effects were verified by a visual inspection.

**Figure 12:**
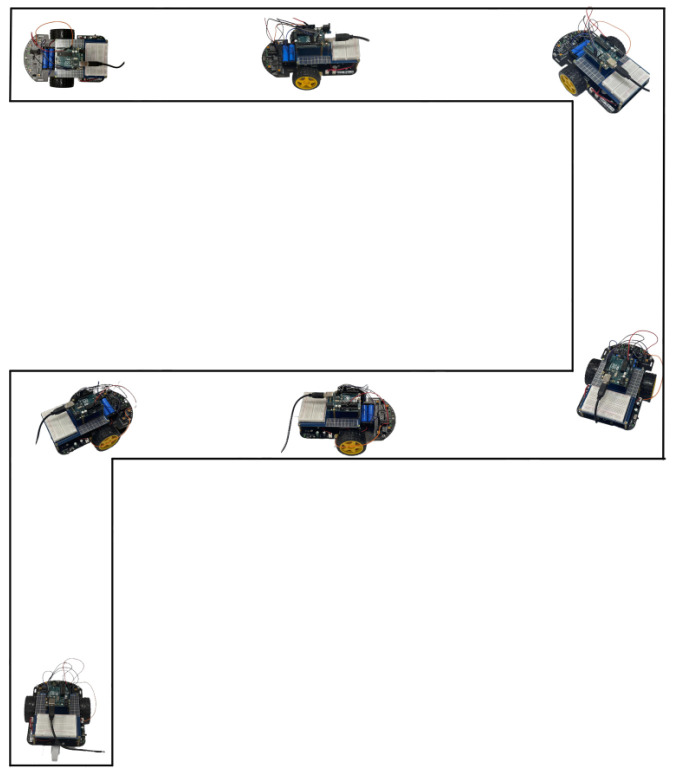
Arduino car movement based on simulated SSVEP.

As shown in Figure 12, the results demonstrated that the classification of simulated SSVEP signals could accurately control the car’s movement direction, successfully completing the preset navigation task.

## 4. Discussion

This study successfully simulated SSVEP signals through the establishment and optimization of a SSVEP-BCI multi-dynamic coupled neural mass model, demonstrating its potential in BCI applications. The results show high consistency between simulated and actual SSVEP signals across multiple frequencies, validating both the effectiveness of the neural mass model in SSVEP signal simulation and providing a theoretical foundation for further BCI technology development.

From the perspective of current research, SSVEP, as a type of EEG signal induced by visual stimulation, shows broad application prospects in brain–computer interface systems due to its high temporal resolution and stability. However, existing SSVEP-BCI technology still faces numerous challenges. While many studies focus on improving SSVEP signal recognition accuracy and stability, challenges, such as individual differences, external stimulus dependency, and experimental environment complexity, remain insufficiently addressed. Our study tackles some of these issues through the introduction of a SSVEP-BCI multi-dynamic coupled neural mass model, utilizing PSO to adjust the model parameters to accommodate individual differences and stimulus frequency characteristics.

Specifically, this research innovates bidirectional coupling modeling of brain regions, simulating interactions between occipital and parietal regions. Compared to the traditional neural mass model, this model more accurately reflects the physiological mechanisms of EEG signal generation, providing a more solid foundation for SSVEP signal recognition and application. Additionally, the use of PSO for weight coefficient optimization across different frequencies effectively enhanced the model’s adaptability to individual differences.

Arduino car control experiments were conducted with the current model under a given sequence of commands, illustrating that simulated SSVEP signals can be used for the manipulation of real devices. Although real experiments require real-time EEG acquisition and BCI parsing and the generation of real-time control commands, experiments under a given command sequence are also valuable. This will facilitate the degree of system simulation emulation of the SSVEP control device, thus enabling BCI system performance testing and optimization without the need to involve real subjects. This approach will allow researchers to rapidly evaluate system performance, test different algorithms and parameter configurations, and validate new BCI designs without the constraints of field experiments. As a result, this approach will not only reduce the cost of research and reduce the reliance on experimental participants but will also increase the reproducibility of research. In addition, simulation modelling will provide researchers with the flexibility to conduct a variety of experiments and scenarios, thus further accelerating the development and application of biometrics.

However, despite the significant advances mentioned above, certain limitations remain. Firstly, the current model only couples the occipital and parietal regions, without considering interactions among more brain areas. Secondly, although PSO improved parameter optimization, model performance may still be limited by the optimization algorithm precision. Additionally, although the data augmentation strategy and FPF-net model are verified for the potential application of our current model, more performance comparisons of the integration of SSVEP simulation and BCI are needed.

Based on our findings, future research could extend in the following directions:

1. Incorporating additional brain regions, such as the prefrontal and temporal areas, into the model. This would involve developing a systematic framework based on the functional connectivity of the cerebral cortex network, thereby enhancing the physiological realism of the model. However, this may also lead to an increase in computational complexity; therefore, future research will need to strike a balance between simulation fidelity and computational efficiency.
2. Investigating the integration of advanced techniques, such as adaptive chaotic PSO or deep reinforcement learning, to further improve the model’s adaptability and robustness in dynamic and complex environments. Additionally, there is also the need to further examine the effects of individual neural system variations on SSVEP signals. This could lead to the development of more refined adaptation strategies, ultimately enhancing the model’s universality and effectiveness for diverse users.
3. Validating the overall performance of the SSVEP-integrated BCI model. To ensure that the generated signal can be used by real SSVEP-BCIs, more performance comparisons need to be conducted to verify if the simulated SSVEP signal is suitable for current algorithms oriented towards real SSVEP signal processing. Whether there are significant differences in performance comparisons under machine learning and deep learning methods in the processing steps, such as preprocessing, feature extraction, and feature classification, needs to be further verified.
4. Extending the model to other non-visual BCI modalities, such as motor imagery- and auditory-based paradigms. However, these signals have different frequency characteristics and dynamic patterns, requiring specific adjustments to the model’s structure, dynamic coupling, and optimized parameters.
5. Extending the model’s applications to a range of devices, including brain-controlled drones, wheelchairs, and smart home systems. This will involve optimizing the mechanisms for controlling multiple devices simultaneously, ensuring precise signal mapping. Moreover, conducting performance validation in dynamic real-world environments also helps to assess the model’s reliability, stability, and practical applicability.

In summary, while this research has achieved significant progress in SSVEP signal modeling and application, numerous challenges remain. Future research will focus on enhancing model accuracy, universality, and compatibility with various brain-controlled devices, promoting the widespread application of SSVEP-BCI technology. These advancements will contribute to making brain–computer interface technology more accessible and practical for real-world applications, ultimately benefiting both research and clinical applications in the field of neural engineering.

## Figures and Tables

**Table 1:** The distribution of SSVEP signals across different frequency bands under different visual stimuli.

Visual Stimulus Frequency	Energy Ratio of Rhythmic Wave Bands		
	0–4 Hz (δ)	8–16 Hz (α)	32–64 Hz (γ)
10 Hz	0.0459	0.1239	0.8303
11 Hz	0.0400	0.1331	0.8269
12 Hz	0.0384	0.1280	0.8336

**Table 2:** Important optimization parameters of PSO algorithm.

Visual Stimulus Frequency	The Coupling Coefficient and Gaussian White Noise Optimization Parameters					
	*o_p_*	*p_o_*	*μ* *_o_*	*σ* ^2^ *_o_*	*μ* *_p_*	*σ* ^2^ *_p_*
10 Hz	1234.18	17.78	1628.27	1709.47	1600.13	7345.33
11 Hz	359.44	64.11	1354.51	17,978.49	1436.16	2261.88
12 Hz	1566.06	370.92	1741.10	4611.35	899.50	7798.72

**Table 3:** Consistency analysis between simulated and real SSVEP Signals.

Visual Stimulus Frequency	Performance Metrics		
	MAE	RMSE	ME
10 Hz	0.3226	0.4182	2.2861
11 Hz	0.1953	0.2458	1.1009
12 Hz	0.1520	0.1906	0.8430

**Table 4:** Accuracy of different classification methods.

	SVM	LSTM	AttnSleep	CMFnet	FPF-Net
**Training set**	0.512	0.731	0.729	0.756	0.821
**Test set**	0.475	0.726	0.714	0.744	0.813

## Data Availability

No new data were created in this study. Data sharing is not applicable to this article.
